# Neuroimaging and Emotional Development in the Pediatric Population: Understanding the Link Between the Brain, Emotions, and Behavior

**DOI:** 10.3390/pediatric17030065

**Published:** 2025-06-08

**Authors:** Giuseppe Marano, Maria Benedetta Anesini, Miriam Milintenda, Mariateresa Acanfora, Claudia Calderoni, Francesca Bardi, Francesco Maria Lisci, Caterina Brisi, Gianandrea Traversi, Osvaldo Mazza, Roberto Pola, Gabriele Sani, Eleonora Gaetani, Marianna Mazza

**Affiliations:** 1 Unit of Psychiatry, Fondazione Policlinico Universitario Agostino Gemelli IRCCS, 00168 Rome, Italy; mbenedetta@hotmail.it (M.B.A.); milintenda@icloud.com (M.M.); mariatacanfora@gmail.com (M.A.); mariannamazza@hotmail.com (M.M.); 2 Department of Neurosciences, Università Cattolica del Sacro Cuore, 00168 Rome, Italy; 3 Unit of Medical Genetics, Department of Laboratory Medicine, Ospedale Isola Tiberina-Gemelli Isola, 00186 Rome, Italy; 4 Spine Surgery Department, Bambino Gesù Children’s Hospital IRCCS, 00168 Rome, Italy; osvaldo.mazza@opbg.net; 5 Section of Internal Medicine and Thromboembolic Diseases, Department of Internal Medicine, Fondazione Policlinico Universitario Agostino Gemelli IRCCS, Università Cattolica del Sacro Cuore, 00168 Rome, Italy; 6 Department of Translational Medicine and Surgery, Fondazione Policlinico Universitario Agostino Gemelli IRCCS, Università Cattolica del Sacro Cuore, 00168 Rome, Italy; 7 Unit of Internal Medicine, Cristo Re Hospital, 00168 Rome, Italy

**Keywords:** neuroimaging, emotions, behavior, children, adolescents, mood

## Abstract

Neuroimaging has emerged as an innovative and essential tool for understanding the intricate relationship between brain development, emotions, and behavior. Investigating the neurobiological mechanisms underlying this interaction during the critical phase of brain maturation is crucial for promoting individual psychological well-being and mitigating the profound impact of mood disorders during childhood. This narrative scoping review synthesizes current pediatric neuroimaging evidence, filling a gap in the literature by integrating structural, functional, and emerging modalities, to provide clear translational pathways for clinical and behavioral observations. The contribution of major neuroimaging techniques, including fMRI, PET, DTI, and sMRI, is analyzed, emphasizing their ability to detect structural and functional alterations associated with mood disorders, enabling early diagnosis and personalized therapeutic strategies. Furthermore, the potential of these technologies to monitor the effects of psychotherapy is explored, demonstrating how such interventions can modulate neural circuits and enhance emotional processing. Despite significant advancements and growing interest, challenges remain, including the complexity of data interpretation, technological limitations, and ethical considerations related to the use of these interventions in pediatric populations. This review synthesizes the most recent scientific evidence, underscoring the potential of neuroimaging to improve diagnostic accuracy and therapeutic outcomes, while outlining future research directions aimed at enhancing interventions for children and adolescents with mood disorders.

## 1. Introduction

Pediatric mood disorders are complex and multifactorial conditions involving disruptions in emotional regulation, cognitive processes, and behavior. These dysfunctions are closely linked to the maturation of specific brain regions and networks, which undergo significant changes during childhood and adolescence.

Emotional development represents a critical aspect of this growth, shaping psychological well-being, social interactions, and individual temperament. Emotions not only drive social behavior but also influence cognitive processes and contribute to overall behavioral regulation. When this developmental process is disrupted, children may experience difficulties in managing emotions, increasing their risk of developing mood disorders such as depression and bipolar disorder [1]. According to the World Health Organization (WHO), approximately 10–20% of adolescents experience mental health disorders [2], with an estimated 6% of those aged 10–19-years affected by mood disorders [3]. Young individuals with mood disorders often face unfavorable long-term outcomes, and the safety of psychotropic medications in this demographic remains a significant concern [4].

In recent years, neuroimaging has emerged as a powerful tool for investigating the intricate interactions between brain development, emotions, and behavior. Advanced techniques, including functional magnetic resonance imaging (fMRI), positron emission tomography (PET), structural magnetic resonance imaging (sMRI), and diffusion tensor imaging (DTI), enable researchers to examine both the structural and functional dynamics of the brain in real time. These methodologies have been pivotal in identifying significant alterations in key brain regions, such as the prefrontal cortex, amygdala, and limbic system, which play a central role in emotional processing and regulation, particularly in children affected by mood disorders, offering valuable insights for early diagnosis and the optimization of therapeutic strategies [5]. These areas undergo profound changes during childhood and adolescence, reflecting the remarkable plasticity of the brain during these developmental stages. However, this same plasticity makes the brain particularly vulnerable to adverse influences, such as genetic predispositions or environmental stressors, which can compromise normal development and lead to emotional dysfunctions.

The application of neuroimaging extends beyond diagnosis. It offers a powerful means of tracking the effectiveness of treatments over time. By visualizing changes in brain structure and function, neuroimaging can provide objective evidence of how various therapies both psychological and pharmacological impact the neural circuits involved in mood regulation [6].

The aim of this review is to provide a comprehensive overview of scientific and clinical advancements in neuroimaging applied to pediatric mood disorders while simultaneously encouraging future research to improve diagnostic and therapeutic strategies.

By promoting targeted and personalized interventions, the goal is to enhance the long-term well-being of young patients. Importantly, this narrative scoping review aims to systematically map clinical, behavioral, and imaging findings across structural, functional, and emerging modalities in pediatric populations, explicitly overcoming the lack of of up-to-date literature on advanced neuroimaging applications for therapeutic monitoring and closed-loop interventions.

## 2. Emotions and Behavior: Brain Areas Involved

**Figure 1:**
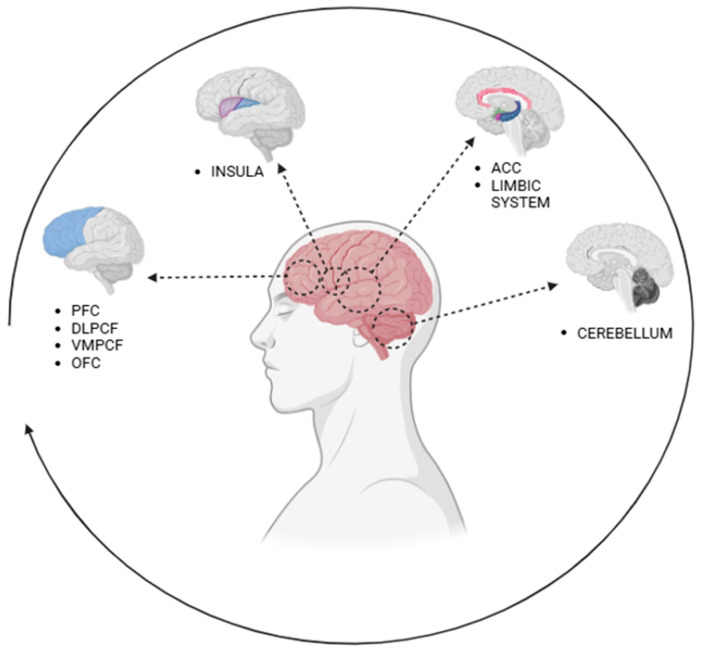
Brain regions involved in the regulation of emotions and behaviors. Note: PFC: prefrontal cortex; DLPCF: dorsolateral PFC; VMPCF: ventromedial PFC; OFC: orbitofrontal cortex; ACC: anterior cingulate cortex.

Emotions and human behavior are complex processes that involve several interconnected brain areas (Table 1). The main brain regions involved in the regulation of emotions and behaviors include the prefrontal cortex, the anterior cingulate cortex, the insula, the cerebellum, and the limbic system, as described in Figure 1 [7].

The prefrontal cortex (PFC), located in the anterior part of the frontal lobes, is the center of emotional and behavioral regulation [8]. Its role in emotion regulation is evident in its control over emotional responses generated by the limbic system, such as the modulation of intense or inappropriate emotions, allowing for adaptive emotional control. It is essential for impulse control, inhibiting automatic or impulsive behaviors to enable thoughtful decision-making and socially appropriate behavior. The PFC is responsible for long-term planning and strategic decision-making, integrating emotional information to assess rewards and risks. It also plays a crucial role in stress regulation, modulating cortisol release and reducing the negative effects of chronic stress [9]. The PFC can be divided into subregions with specific functions. The dorsolateral prefrontal cortex (DLPFC) is involved in cognitive control, working memory, and planning, integrating emotional information with complex decision-making processes. The ventromedial prefrontal cortex (VMPFC) is crucial for emotional processing and value- and reward-based decision-making, with strong interactions with the amygdala, nucleus accumbens, and hippocampus [10]. The orbitofrontal cortex (OFC), connected to the dorsolateral prefrontal circuit, is involved in the manipulation and integration of sensory information from the external environment via the temporal cortex and the insula. In addition, it is implicated in mediating empathetic and socially appropriate responses by receiving information from the internal environment, with strong connections to the anterior cingulate cortex (ACC) [11]. In anxiety disorders, insufficient control over the amygdala leads to exaggerated fear responses [12]. Dysfunction of the OFC can lead to loss of control and compulsive behaviors [13,14]. In attention deficit hyperactivity disorder (ADHD), dysfunction of the DLPFC is associated with attention and self-control problems [15]. The cerebellum, traditionally associated with motor control, movement coordination, and balance, also plays a role in emotion processing and behavior regulation [16]. Recent studies have highlighted that this structure, through its connections with other brain areas, contributes to emotional identification and expression, as well as expression related to empathy [17]. Lesions in the posterior lobe result in cerebellar cognitive affective syndrome (CCAS), which includes deficits in executive function, visuospatial processing, language abilities, and affect regulation [18]. Anomalies in cerebello-limbic connections have been associated with maladaptive behaviors and social problems [19,20]. The insula is a region of the cerebral cortex located deep within the lateral sulcus, hidden between the temporal, parietal, and frontal lobes. It plays a fundamental role in integrating sensory, emotional, and cognitive signals. The insula monitors the body’s internal states, such as heartbeat, hunger, and body temperature, contributing to bodily awareness [21]; it regulates the subjective experience of emotions such as disgust, fear, empathy, and pleasure [22]; it plays a key role in the perception of physical and emotional pain and is involved in social suffering, such as rejection or loss [23,24]. The anterior insula is particularly active in empathy processes and in understanding others’ emotions, while the posterior insula is more related to sensory perception and visceral regulation [25,26,27]. This structure is also involved in addiction and compulsive behavior, contributing to the awareness of craving states such as the desire for drugs, food, or cigarettes [28,29,30]. It also plays an important role in decision-making and intuition, helping to connect bodily experience to choices. The limbic system is a set of brain structures involved in emotion processing, memory, and motivation and includes the amygdala, the hippocampus, the cingulate gyrus, the hypothalamus, and the nucleus accumbens [31].

The amygdala is an almond-shaped structure located in the limbic system, crucial for the perception and regulation of emotions [32]. The amygdala is responsible for processing emotionally salient stimuli and modulating behavioral responses based on the emotions perceived [33]. This crucial role manifests in several aspects. The amygdala detects emotional stimuli, quickly identifying signals of threat or emotional relevance in the environment, such as an angry person’s face or a dangerous situation. This automatic process prepares the organism to respond appropriately. Through its connection with other brain areas, such as the hypothalamus and brainstem, the amygdala coordinates behavioral and physiological responses, such as activating the “fight or flight” system during stressful or dangerous situations [34]. The amygdala interacts with the hippocampus to store memories associated with strong emotions, such as traumatic or significant events [35]. These emotional memories subsequently influence behavior, guiding the individual to avoid situations perceived as threatening or to repeat rewarding ones [36]. The amygdala works with the PFC to regulate the emotional response based on context. While the amygdala generates rapid emotional reactions, the PFC exerts top-down control, helping to modulate and adapt emotional behaviors to complex or socially appropriate situations [37]. Dysfunctions in amygdala activity can lead to altered emotional behaviors, such as impulsive aggression or chronic anxiety. Excessive activation can amplify inappropriate behavioral responses, while reduced activity can impair risk perception. Overall, the amygdala acts as a bridge between emotions and behaviors, transforming emotional perception into concrete responses. Its interaction with other brain structures ensures a balance between rapid reactions and contextual regulation, which is essential for adaptive behavior and survival. The ACC, located in the anterior portion of the gyrus, serves as a bridge between the limbic system, responsible for emotions, and the prefrontal cortex, involved in higher cognitive processes and behavior regulation [38]. The ACC is central to emotional regulation, monitoring and modulating emotional responses generated by the amygdala. Through connections with the limbic system, the ACC helps regulate the intensity of emotions such as fear, anxiety, and anger, playing a key role in reducing excessive emotional activation. A crucial aspect of the ACC is its contribution to error monitoring and conflict resolution [39]. When a behavior does not produce the expected outcome, the ACC is activated to detect discrepancies and direct attention to the error, promoting learning and adaptation. This mechanism is essential for behavioral control and maintaining motivation, especially in contexts of uncertainty or difficulty. The ACC is also involved in the management of both physical and emotional pain [40]. Its activation is associated with the processing of social pain, such as rejection or exclusion, as well as physical pain. This highlights its role in linking bodily experiences and emotional states, contributing to a coherent behavioral response. In social interactions, the ACC plays a role in regulating empathy and prosocial behavior, and helps us to understand others’ emotions, integrating social cues and emotional information to promote appropriate responses and support interpersonal connection [41]. In depression, reduced activity in the ACC can impair emotional regulation and the ability to cope with stressful events [42]. In schizophrenia, dysfunctions of the ACC are linked to issues with behavioral control and the inability to integrate emotional and cognitive information [43,44]. The hippocampus, also located in the temporal lobe, is crucial for the formation of long-term memory and learning [45]. It plays an important role in contextualizing emotions, associating specific events with emotional stimuli [46,47]. This function is essential for understanding and regulating appropriate behavioral responses based on past experiences, such as in anxiety disorders [48]. The hypothalamus plays a role in coordinating physiological responses to emotions: it regulates autonomic functions such as heart rate, blood pressure, and hormone release through the hypothalamic–pituitary–adrenal axis [49,50]. It is essential for maintaining homeostasis and modulating fundamental behaviors such as aggression, hunger, and mating [51].

The nucleus accumbens is involved in the perception of pleasure and the processing of rewards [52,53]. Its connections with the dopaminergic system make it crucial for behaviors related to the pursuit of gratification and for the regulation of addiction [54,55].

The limbic system integrates emotions, memory, and behaviors, allowing for adaptive responses to the demands of the environment. However, imbalances or dysfunctions in these structures can lead to emotional and behavioral difficulties, such as anxiety, depression, or uncontrolled aggression. 

## 3. The Neuroplasticity of the Pediatric Brain

The neuroplasticity of the pediatric brain, or its ability to adapt and reorganize in response to experiences, environmental stimuli, and learning, is a fundamental aspect of child development [56,57]. Unlike the adult brain, which is more stable, the brain of a child has an incredible ability to reshape itself, making it highly adaptable but also vulnerable to external influences [58,59,60]. Brain plasticity in children is one of the most remarkable characteristics of the developing brain. During childhood, the brain has an exceptional ability to reorganize itself in response to environmental stimuli, strengthening or eliminating synaptic connections based on experience and in response to congenital or traumatic neurological conditions [61,62,63,64]. Siffredi et al. demonstrated that neuroplasticity in children with corpus callosum agenesis appears to allow functional connectivity comparable to that of a typically developing brain, increasing intra-hemispheric connectivity [65]. Childhood is characterized by high synaptic density and greater neural flexibility, enabling children to rapidly acquire new skills such as language, motor abilities, and cognitive functions, adapt to their environment, and modulate their emotional responses [66,67,68,69]. Emotional responses in children are profoundly influenced by synaptic plasticity, which shapes the neural circuits responsible for affective regulation [70,71]. During the early years of life, emotional experiences, both positive and negative, shape brain connectivity, influencing how a child will respond to stress, reward, and social interactions [72,73]. We differentiate between infancy (synaptogenesis peak), early childhood (pruning and language/emotion circuit refinement), and adolescence (pubertal maturational surge in limbic–PFC connectivity), each with distinct vulnerabilities and therapeutic windows [74].

**Figure 2:**
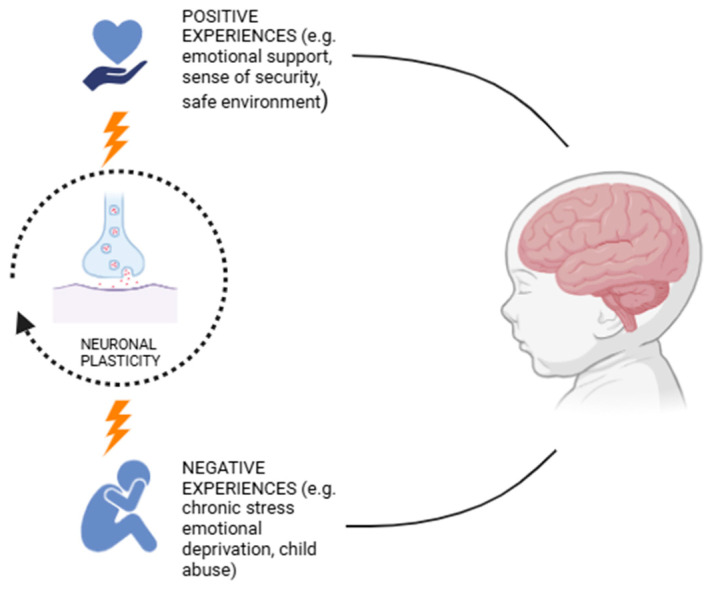
Effect of emotional experiences on neuroplasticity.

Sensory, emotional, and cognitive experiences actively shape the structure and function of a child’s brain. Plasticity allows for a high degree of adaptability, supporting the development of effective emotional regulation strategies and the ability to recognize and interpret both one’s own emotions and those of others [75,76]. Positive experiences, such as emotional support and a sense of security, help build resilient neural circuits, foster emotional stability, and reduce susceptibility to mood disorders [77]. Conversely, exposure to chronic stress or emotional deprivation can lead to altered synaptic plasticity, increasing the risk of anxiety, depression, and difficulties in emotional regulation, as described in Figure 2 [78,79,80,81,82,83]. Self-dissatisfaction can influence brain neuroplasticity, which, in turn, affects self-perception [84]. Induced neuroinflammation, for example, from COVID-19, could alter microglial cell function, impairing brain plasticity and contributing to learning difficulties and neurodevelopmental disorders. This occurs through a reduction in brain-derived neurotrophic factor (BDNF), alteration an in communication between immune cells and microglia, increased inflammatory molecules, and the disruption of crucial signaling pathways for synaptic plasticity [85,86]. However, brain plasticity in childhood also offers recovery opportunities, enabling targeted therapeutic interventions to strengthen neural connections related to emotional well-being [87]. A key aspect of childhood plasticity is its compensatory ability [88,89]. If a brain region responsible for emotional regulation suffers a deficit, other regions may step in to support these functions, making effective adaptation possible. This property is essential for early interventions aimed at children with emotional difficulties, allowing the modulation of responses through educational and therapeutic strategies based on neuroplasticity. After the early years of life, neuroplasticity begins to slow down [90]. This property is essential for early interventions aimed at children with emotional difficulties, allowing emotional responses to be modulated through educational and therapeutic strategies based on neuroplasticity.

## 4. Role of Neuroimaging in the Study of Mood Disorders

Neuroimaging has become an indispensable tool to investigate the neurobiological mechanisms underlying mood disorders, especially given that these disorders are a leading cause of disability worldwide. By providing a non-invasive, real-time window into brain structure and function, brain imaging techniques have helped us to understand how brain networks are involved in emotion regulation, cognitive control, and mood stability development and function in both adults and children, including adolescents [91,92]. These imaging techniques not only aid in the detection of early biomarkers, but also allow clinicians to monitor disease progression, to assess the efficacy of treatments, and to develop more personalized therapeutic interventions. The imaging techniques available in clinical settings include the following: magnetic resonance (MRI), which assessed anatomy (structural MRI) and brain activity (functional MRI); positron emission tomography (PET), which evaluates brain metabolism and neurotransmitter activity; and diffusion tensor imaging (DTI), which examines the brain’s white matter connections (Table 2).

Structural, or volumetric, neuroimaging has been used to investigate the correlation between pathophysiology and measurable changes in neuroanatomy, identifying several anatomical abnormalities in brain regions that are thought to affect regulation and emotional expression.

MRI generates images through the differential magnetic properties of hydrogen in different tissues.

Structural MRI (sMRI), which provides high-resolution anatomical imaging, has been instrumental in identifying structural abnormalities associated with mood and behavioral disorders. Studies have reported reductions in gray matter volume in key emotion-regulating regions such as the prefrontal cortex, hippocampus, and amygdala, with these deficits being more pronounced in individuals with early-onset depression and recurrent mood disorders [93,94].

Functional MRI (fMRI) detects brain activity through blood-oxygen-level-dependent (BOLD) signals, making it possible to map functional networks involved in emotional regulation. A different component of hydrogen atoms is measured which reflects the ferromagnetic nature of deoxygenated vs. oxygenated blood. Thus, an area with more oxygenated blood will appear more intense compared to when there is less oxygenated blood.

Research has consistently shown hyperactivity in the amygdala and reduced connectivity between the prefrontal cortex and limbic system in children with depression, highlighting mechanisms that contribute to emotional dysregulation and heightened sensitivity to negative stimuli [95].

PET imaging, through the use of radioactive neurotransmitter derivatives, produces a three-dimensional image of the brain, allowing the study of metabolic activity and neurotransmitter function, thereby clarifying the neurochemical imbalances in mood disorders. Findings from PET studies, for example, have linked pediatric depression to serotonin and dopamine dysregulation, as well as reduced glucose metabolism in the DLPFC, OFC, PFC, and CCA—patterns that correlate with symptoms such as low motivation, cognitive dysfunction, and emotional blunting [96,97].

DTI, an advanced MRI-based technique, is crucial for mapping white matter pathways and evaluating brain connectivity. By examining disruptions in major tracts such as the corpus callosum, uncinate fasciculus, and cingulum bundle, DTI has provided a valuable insight into BD, where abnormal connectivity is thought to contribute to mood instability, impulsivity, and cognitive deficits [98,99,100,101]. Together, these neuroimaging techniques offer a comprehensive view on the neural basis of emotional development in children and adolescents, leading the way for earlier diagnosis, targeted treatments, and a deeper understanding of pediatric mood disorders.

## 5. Psychotherapy and Neuroimaging

In recent decades, functional neuroimaging techniques have revolutionized our understanding of the neural mechanisms underlying psychiatric disorders. More recently, their use has expanded to psychotherapy, allowing not only for the confirmation of brain changes induced by psychotherapeutic interventions but also for the development of new approaches based on neural biomarkers. The integration of neuroimaging into clinical practice is transforming psychotherapy from a model based solely on symptoms and behaviors to a biologically informed approach, in which modifications in brain networks can be used to monitor treatment response and personalize therapeutic interventions.

A growing body of research has demonstrated that psychotherapy does not merely modify behavior and cognition but also induces measurable neurobiological changes. Among the most extensively studied approaches, cognitive behavioral therapy (CBT) has been shown to have a significant impact on brain connectivity. In particular, it has been observed that in patients with anxiety disorders, CBT strengthens prefrontal control over subcortical structures involved in fear regulation, such as the amygdala and ACC, thereby contributing to better modulation of emotional responses [102]. This effect extends to mood disorders as well, where activation of the subgenual ACC has been found to predict treatment efficacy: elevated metabolic activity in this region has been associated with poorer therapy response, highlighting the need for more targeted therapeutic approaches [103].

The use of neuroimaging to monitor the effects of psychotherapy is also expanding to the pediatric population, particularly in the study of mood and anxiety disorders. One study examined the association between reward circuit function and psychotherapy response in young individuals with anxiety disorders, revealing that higher pre-treatment activation in the medial prefrontal cortex and nucleus accumbens was correlated with better therapy outcomes. These findings suggest that neuroimaging could serve as a tool to identify young patients who are more likely to benefit from psychotherapeutic interventions [104].

In the context of major depressive disorder (MDD), CBT has been shown to induce significant modifications in brain connectivity. A study on adolescents with remitted MDD found that rumination-focused cognitive behavioral therapy (RF-CBT) not only improved clinical symptoms but also induced changes in neural circuits involved in rumination regulation, such as the left precuneus and angular gyrus. Additionally, the low stability of brain activations in treated patients suggests that therapy does not merely reinforce existing circuits but promotes the dynamic reorganization of neural networks involved in repetitive thinking [105]. Other studies have demonstrated that CBT can enhance connectivity between the subgenual ACC, the amygdala, and frontal regions, suggesting that the treatment does not simply normalize brain activity but contributes to the functional readjustment of neural networks in MDD patients [106].

Another key aspect concerns the interaction between sleep, emotional regulation, and neurobiology. Insomnia is a known risk factor for depression, and neuroimaging studies have shown that targeted interventions can significantly mitigate this risk. In particular, CBT for insomnia (CBT-I) combined with circadian rhythm support (CRS) has been found to enhance amygdala response and improve reactivity in the left insula, both crucial regions for emotional regulation [107].

Alongside CBT, mindfulness and meditation-based interventions are emerging as complementary tools for emotional regulation, with distinct neurobiological effects. Notably, mindfulness-based cognitive therapy for children (MBCT-C) has shown improvements in brain connectivity and emotional regulation in youth with a familial risk of bipolar disorder, suggesting a positive impact on resilience [108]. The efficacy of mindfulness has also been confirmed by the Mindfulteen study, which demonstrated that mindfulness can reduce stress reactivity and anxiety in adolescents by modulating connections between the PFC and the limbic system [109].

The benefits of meditation have been further validated by studies examining brain connectivity in adolescents with major depression. Body–mind relaxation meditation (BMRM) has been shown to normalize alterations in thalamocortical connectivity, with positive effects on attention and self-referential processes [110]. Additionally, mindfulness has been found to increase gray matter volume in the right hippocampus, a crucial region for memory and stress regulation, with significant benefits in reducing depressive symptoms and perceived stress in individuals with a history of childhood maltreatment [111].

Finally, a pilot study showed that a mindfulness-based intervention (MBI) increased connectivity between the PCC and the DLPFC, regions involved in emotional regulation. This change was associated with reduced suppression of negative emotions and increased awareness, suggesting that these practices could serve as a therapeutic target for mood stabilization in at-risk youth [112].

Overall, these findings confirm that neuroimaging not only allows for the evaluation of psychotherapy’s direct effects on brain circuits, but is also emerging as a key tool for optimizing therapeutic interventions. The identification of neurofunctional biomarkers could enable the personalization of treatment selection and the prediction of the likelihood of success for a given psychotherapeutic approach. While CBT remains an effective strategy for reshaping brain connectivity in patients with anxiety and depression, mindfulness and meditation practices are emerging as complementary strategies for improving emotional regulation and enhancing resilience. The integration of neuroscience and psychotherapy could therefore represent a promising avenue for improving the efficacy and precision of therapeutic interventions.

## 6. Role of Neuroimaging and Emotion Regulation in Adolescence: A Window into the Developing Brain

Modern neuroimaging techniques, including fMRI, magnetic resonance spectroscopy (MRS), and PET, provide an advanced means of investigating structural and functional abnormalities in the brain. These methods are particularly valuable in studying younger populations, where the brain is still undergoing critical developmental processes. By enabling researchers to visualize neural activity, connectivity, and biochemical composition, these imaging modalities serve as a powerful tool for examining how different brain regions interact, activate, and, in some cases, exhibit dysfunctions linked to psychiatric disorders. This approach not only deepens our understanding of neurodevelopmental and psychiatric conditions but also supports the development of more targeted therapeutic interventions.

Adolescence is a critical period of emotional vulnerability due to the ongoing maturation of emotion regulation mechanisms and the development of self-identity [113]. Emotion regulation encompasses the process through which individuals modulate their emotional responses, determining which emotions they experience and when they occur. The immaturity of neural networks responsible for emotion regulation may contribute to the onset and exacerbation of mood disorders, as adolescents often struggle to sustain positive emotions while exhibiting an exaggerated response to negative stimuli. Impairments in these regulatory mechanisms, along with the reliance on maladaptive regulatory strategies, are linked to various psychiatric disorders and play a significant role in their persistence [114].

This heightened emotional sensitivity increases the risk of early-onset mood disorders, which are often associated with greater symptom severity [115] and a poorer long-term prognosis [116]. Moreover, adolescent depression is strongly linked to an elevated risk of suicide, emphasizing the need for early and targeted interventions. The combination of impaired emotion regulation and a tendency toward self-focused rumination further exacerbates vulnerability to depression and increases the risk of suicide attempts [117].

Recent advancements in brain imaging techniques have greatly enhanced the identification of neural markers linked to mood disorders in children and adolescents. Early detection of these conditions is essential for ensuring timely interventions and improving long-term outcomes. Advanced imaging methods allow for the identification of brain biomarkers that may indicate a predisposition to mood disorders even before the onset of overt clinical symptoms, providing valuable insights into early pathological changes and potential targets for preventive strategies.

A consistently observed finding is the reduced functional connectivity between the amygdala and the PFC, a pattern associated with impaired emotion regulation and heightened vulnerability to depression in young individuals. Neurodevelopmentally, adolescence is characterized by increased activity in limbic structures, particularly the amygdala, while the medial prefrontal cortex (mPFC), essential for regulating emotions, remains underdeveloped [118]. Consequently, adolescents experience emotions with greater intensity due to reduced top-down regulatory control by the mPFC [119].

Given these neurodevelopmental dynamics, understanding the neural basis of emotion regulation in adolescents is crucial for developing targeted interventions that mitigate the risk of mood disorders.

In this context, emotion regulation has become a primary focus in real-time functional magnetic resonance imaging neurofeedback (rt-fMRI-NF) research, a promising tool for enhancing emotion regulation capacities [120]. This method shows potential in achieving long-term symptom improvement in psychiatric conditions such as post-traumatic stress disorder (PTSD) [121], borderline personality disorder (BPD) [122], and attention deficit hyperactivity disorder (ADHD) [123]. Empirical studies indicate that healthy individuals can actively modulate their brain activity in response to rt-fMRI-NF across multiple regions implicated in emotion regulation, such as the amygdala, anterior insula, and ACC. Furthermore, brain activity regulation via rt-fMRI-NF has been demonstrated to be feasible in modulating both prefrontal–limbic connectivity and specific, individually targeted brain regions [120]. These findings highlight the potential of rt-fMRI-NF as a novel intervention for enhancing emotion regulation, particularly in vulnerable populations such as adolescents. Early identification of at-risk individuals is essential to implementing preventive and therapeutic strategies that may mitigate long-term consequences. In this context, neuroimaging has emerged as a powerful tool for detecting structural and functional brain alterations linked to mood disorders before clinically evident symptoms appear. It has also facilitated the identification of biomarkers associated with pediatric bipolar disorder (PBD), enhancing early diagnosis and treatment precision. Numerous neuroimaging studies have documented neuroanatomical abnormalities in PBD, though findings remain inconsistent, likely due to the intrinsic complexity and heterogeneity of the disorder itself [124]. The study by Otten and Meeter [125] found that bipolar disorder (BD) is associated with a reduction in hippocampal volume, particularly in early-onset cases. These findings suggest that hippocampal abnormalities may contribute to BD pathophysiology from its earliest stages, offering important insights for early intervention and targeted therapies. Another study by Kiani et al. [126] has identified notable white matter alterations in PBD, particularly a reduction in fractional anisotropy (FA) within the ACC, anterior corona radiata, and the genu of the corpus callosum, suggesting a link between these structural changes and clinical symptoms. Additionally, the uncinate fasciculus demonstrated atypical developmental patterns, emphasizing its potential role in the disorder. In terms of structural connectivity, graph analysis revealed extensive disruptions, particularly in the OFC, frontal gyrus, and basal ganglia. A key finding was the weakened connectivity between the prefrontal and limbic regions, which may underlie the emotional instability frequently observed in PBD. These structural and connectivity alterations could serve as potential neurobiological biomarkers, aiding in early diagnosis, risk assessment, and the development of targeted therapeutic interventions.

For children and adolescents with mood disorders that do not respond to conventional treatments, advanced brain imaging techniques are proving to be invaluable for refining therapeutic strategies and improving clinical outcomes. By offering a deeper understanding of the neural circuits involved in these conditions, it enables more precise and personalized interventions.

One significant application is in targeting dysfunctional neural pathways. By identifying the specific brain regions associated with symptoms, these imaging techniques help guide neuromodulatory treatments such as repetitive transcranial magnetic stimulation (rTMS), which may restore activity in affected areas and offer relief for adolescents with treatment-resistant depression [127].

Another major advantage is optimizing pharmacological interventions [128]. By identifying biomarkers predictive of treatment response, neuroimaging assists clinicians in selecting the most suitable medication, reducing the time spent on ineffective therapies and lowering the risk of adverse effects. This precision medicine approach moves psychiatric treatment toward a model where interventions are tailored to individual neurobiological profiles.

Moreover, neuroimaging is advancing the development of innovative therapies. Research has supported the use of ketamine for severe depression in young patients, and brain imaging may help identify individuals who are most likely to benefit from this treatment [129]. Unlike traditional antidepressants, which can take weeks or months to exert their effects, ketamine has been shown to induce rapid improvements in mood and suicidality, with some patients experiencing significant changes within just a few hours after administration, sometimes as early as four hours post-treatment [130]. By integrating neuroimaging into clinical practice, healthcare providers can design more effective and individualized treatment plans, offering new possibilities for children and adolescents struggling with mood disorders that do not respond to standard care.

Brain imaging has become an essential tool in the follow-up of pediatric patients with mood disorders, offering valuable insights into the course of the illness and treatment response. By detecting subtle changes in brain activity before symptoms reappear, it allows for the early identification of relapse risk, enabling timely interventions to prevent full recurrence [131]. This proactive approach can improve long-term outcomes and reduce the burden of chronic mood instability. Moreover, advanced imaging techniques provide an objective assessment of treatment effectiveness, helping clinicians evaluate how the brain responds to different pharmacological and psychotherapeutic interventions. Beyond monitoring treatment response, brain imaging also enhances our understanding of the neurobiological factors that contribute to disease persistence [132]. Identifying these underlying mechanisms can lead to the development of personalized prevention strategies, minimizing the risk of long-term impairment.

While conventional MRI and PET approaches have laid the foundation for pediatric neuroimaging, several advanced techniques promise to deepen our understanding of brain–behavior relationships and enable earlier, more precise clinical interventions.

In addition to fMRI, PET, DTI, and sMRI, emerging techniques such as magnetoencephalography (MEG), magnetic resonance spectroscopy (MRS), high-density EEG with source localization, and hybrid MR-PET systems are gaining traction in pediatric research. MEG offers millisecond temporal resolution of circuit dynamics; MRS quantifies neurochemical changes (e.g., GABA, glutamate); EEG source localization can non-invasively track functional connectivity in free-behaving children; and MR-PET combines molecular and structural imaging for unparalleled insight into neurotransmitter metabolism. By integrating these emerging modalities, each with its unique strengths in temporal resolution, biochemical specificity, or hybrid imaging potential, researchers can construct a richer, multimodal portrait of pediatric brain development. Even the brief inclusion of representative plates or example studies for MEG, MRS, EEG source maps, and MR-PET underscores the field’s trajectory toward truly comprehensive, translational neuroimaging.

**Figure 3:**
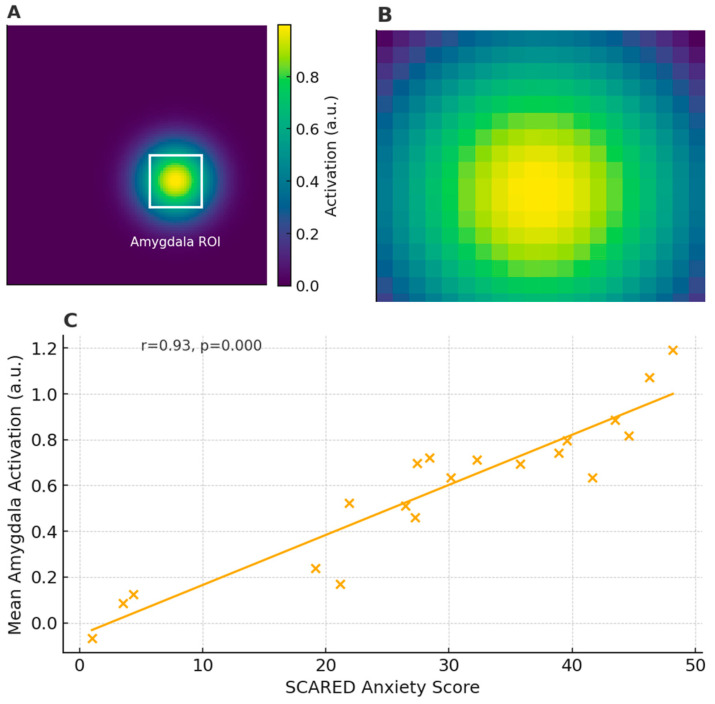
Example of task-based pediatric fMRI activation map: (**A**) Pediatric fMRI activation map overlaid on pediatric template; white box indicates amygdala ROI (Region of Interest). (**B**) Zoomed-in view of amygdala ROI. (**C**) Scatterplot of mean amygdala activation vs. SCARED (Screen for Child Anxiety-Related Emotional Disorders) anxiety scores.

Representative plates are high-quality images, often arranged as multi-panel figures, that serve as visual exemplars of the key methods, findings, or analytical steps used to link brain structure/function with clinical or behavioral measures. They may facilitate understanding the imaging modality (e.g., what an fMRI activation map looks like in children performing an emotion-processing task), clarifying the definition of regions or pathways (e.g., overlays of an anatomical atlas on structural images), and appreciating the correlation between imaging metrics and clinical/behavioral scores (e.g., scatterplots of fractional anisotropy vs. attention performance). In other words, representative plates can be considered visual examples of how neuroimaging data are acquired, processed, and statistically linked to meaningful clinical and behavioral endpoints in pediatric populations. Figure 3 presents an example of a task-based pediatric fMRI activation map.

In this figure, the amygdala ROI (Region of Interest) refers to a rectangular zone placed over the amygdala, which plays a key role in processing emotions such as fear and anxiety. By averaging the BOLD (blood-oxygen-level-dependent) signal within this ROI, researchers obtain a single value (e.g., mean activation intensity) that reflects how strongly the amygdala responds during the task across each subject. This approach reduces noise from other brain regions and allows a direct comparison between subjects or conditions. The Screen for Child Anxiety-Related Emotional Disorders (SCARED) is a standardized, parent- and/or self-report questionnaire used to assess symptoms of anxiety in children and adolescents. It consists of 41 items across five subscales (panic/somatic, generalized anxiety, separation anxiety, social phobia, and school phobia), each rated on a three-point scale (0 = “Not True or Hardly Ever True”, 1 = “Somewhat True or Sometimes True”, 2 = “Very True or Often True”). The total scores range from 0 to 82, with higher scores indicating greater anxiety symptomatology. In the scatterplot (Panel C), each child’s total SCARED score is plotted against their mean amygdala activation, allowing us to see how neural reactivity relates to clinically assessed anxiety levels. A strong positive correlation (r = 0.93, *p* < 0.001) suggests that children reporting more anxiety symptoms also exhibit higher amygdala responses during the emotional-processing task.

## 7. Limitations in Neuroimaging Techniques

Despite the significant advancements in neuroimaging, its application in studying the relationship between brain activity, emotions, and behavior remains complex. While these techniques provide valuable insights into which brain regions are activated during emotional processing, the connection between neural activity and subjective emotional experience is not always straightforward. Emotions emerge from intricate interactions between multiple brain networks, and their expression can vary depending on context, individual differences, and environmental influences.

Interpreting neuroimaging data is further complicated by the fact that changes in brain activity in specific regions may not be exclusive to a single psychiatric condition or cognitive process. For instance, alterations observed in areas such as the prefrontal cortex, amygdala, or hippocampus may be associated with multiple disorders, including anxiety, depression, or bipolar disorder. This overlapping neural signature makes it difficult to determine whether observed changes are disorder-specific or reflect broader variations in brain function [133].

Another major challenge is differentiating between normal neurodevelopment and pathological alterations. The pediatric brain is highly plastic and continuously evolving, with structural and functional modifications occurring naturally during growth. Some brain changes identified in neuroimaging studies may reflect typical developmental processes rather than early markers of psychiatric disorders. As a result, establishing clear-cut criteria to distinguish between normative and pathological brain alterations remains a significant limitation in pediatric research [134].

Moreover, sample size limitations and population heterogeneity present challenges for the robustness and generalizability of findings. Many neuroimaging studies involve small or clinically diverse samples, leading to variability in results and difficulties in replicating findings across different populations. Additionally, psychiatric conditions are often heterogeneous, with symptoms manifesting differently among individuals, further complicating efforts to establish universal neural biomarkers [135].

Beyond biological factors, socio-cultural and environmental influences play a critical role in shaping brain development and mental health. However, these variables are often underrepresented or inadequately controlled in neuroimaging studies. Differences in socioeconomic status, educational background, early-life stress, and cultural upbringing can significantly impact both brain function and mental health outcomes, yet their effects are frequently overlooked. This selection bias limits the applicability of findings to broader populations and may introduce confounding factors that affect the interpretation of results.

Additionally, technological and methodological constraints pose further obstacles, particularly when studying young children. Techniques such as fMRI require participants to remain still for extended periods, which can be particularly challenging for young children or those with mood disorders and emotional dysregulation. Motion artifacts, compliance difficulties, and variations in attentional capacity can reduce the reliability of neuroimaging data in pediatric populations, making it difficult to draw definitive conclusions [136].

While neuroimaging remains an invaluable tool for advancing our understanding of the neural mechanisms underlying emotion and behavior, these methodological and conceptual challenges highlight the need for multidisciplinary approaches, improved study designs, and larger, more representative samples. Addressing these limitations is essential for ensuring that neuroimaging findings translate into meaningful clinical applications, particularly in the early identification and treatment of psychiatric disorders in children and adolescents.

## 8. Conclusions and Future Directions

Neuroimaging has become an essential tool for exploring the intricate relationship between brain development, emotional regulation, and mood disorders in children and adolescents. Research has consistently demonstrated structural and functional changes in key neural circuits, including the prefrontal cortex, amygdala, and limbic system, shedding light on the neurobiological underpinnings of emotional dysregulation. These discoveries have paved the way for early diagnosis and more tailored therapeutic approaches, enhancing treatment strategies for young patients with mood disorders.

However, several challenges remain. The interpretation of neuroimaging data is complicated by individual differences in brain maturation, the overlapping characteristics of various psychiatric conditions, and technical limitations such as motion artifacts in pediatric studies. To overcome these hurdles, future research should prioritize the integration of multiple neuroimaging techniques, leverage Artificial Intelligence for more refined data analysis, and conduct large-scale longitudinal studies to better capture the evolving nature of brain development.

Moving forward, the field offers exciting possibilities. The combination of advanced neuroimaging with Artificial Intelligence (AI)-driven analytics may enhance diagnostic accuracy and help identify early biomarkers for mood disorders. Additionally, innovative treatment models, such as real-time neurofeedback and personalized interventions, hold promise for improving clinical outcomes. Building on identified gaps, we propose (1) longitudinal multimodal imaging studies to chart normative and pathological trajectories; (2) AI-driven analytics for individualized biomarker discovery; (3) the integration of real-time neurofeedback into clinical protocols; (4) standardized pipelines for combining nutrition/gut–brain axis assessments with brain imaging; and (5) pediatric-specific ethical frameworks and trial designs to accelerate closed-loop interventions.

Conducting prospective, repeated-measures studies that combine structural MRI, diffusion tensor imaging (DTI), functional MRI (task-based and resting-state), and even PET or MR spectroscopy allows us to chart both normative brain development (e.g., synaptic pruning trajectories from infancy through adolescence) and the divergent paths seen in children who go on to develop mood or anxiety disorders. For example, leveraging cohorts like the NIH MRI Study of Normal Brain Development alongside specialized clinical samples can help disentangle age-related changes in prefrontal-amygdala connectivity from disease-related alterations [137].

Machine learning frameworks, ranging from random forests and support vector machines to deep learning convolutional neural networks, can ingest high-dimensional imaging and behavioral data to uncover latent phenotypes or “brain signatures” predictive of treatment response. For instance, recent studies have shown that gradient-boosted decision trees applied to DTI metrics can identify children at highest risk for persistent anxiety with >85% accuracy [138]. Embedding these algorithms within clinical workflows could one day enable “precision neuropsychiatry,” where each child’s unique connectivity profile informs a targeted intervention plan.

Real-time fMRI neurofeedback (rt-fMRI-NF) and EEG-based neurofeedback platforms allow children to see, in seconds, how their own brain activity (e.g., amygdala or prefrontal cortex activation) changes as they engage in emotion regulation strategies. Pilot trials in adolescents with major depressive disorder demonstrate that training to down-regulate amygdala hyperactivity via rt-fMRI-NF can produce sustained mood improvements at follow-up [139]. Embedding these protocols into existing cognitive behavioral therapy sessions may enhance skill acquisition and long-term resilience.

Robust evidence links early-life nutrition and microbiota-derived neuroactive metabolites to myelination, neurotransmitter synthesis, and emotional regulation. Deficiencies in iron or docosahexaenoic acid (DHA) impair white matter integrity and synaptic plasticity, increasing the risk for mood and attention disorders. The bidirectional gut–brain axis modulates neuroinflammation and HPA-axis activity, suggesting that combined dietary and microbiota-targeted interventions might serve as effective adjunctive therapies in pediatric mood disorders. Nutrition and the gut microbiome critically shape neurodevelopment: iron and omega-3 fatty acid deficiencies impair myelination, while microbial metabolites like short-chain fatty acids modulate neuroinflammation. We advocate for harmonized protocols that collect dietary records, blood biomarkers (e.g., ferritin, DHA levels), stool microbiome sequencing, and multimodal imaging in the same participants. Such pipelines, exemplified by the emerging “NutriBrain” project, will permit direct correlations between nutrient status, microbial diversity, and imaging metrics like fractional anisotropy or functional connectivity [140,141,142].

Children require special protections in neuroimaging research, such as minimizing sedation, limiting radiation exposure (for PET), and securing both parental consent and age-appropriate assent. It is necessary to recommend developing standardized ethical guidelines that address data privacy (especially as AI analytics become more powerful), the minimal-risk threshold for repeated scans, and culturally sensitive consent processes. Furthermore, adaptive trial designs (e.g., Bayesian or N-of-1 trials) can tailor intervention parameters, such as the neurofeedback target region or dietary supplement dose, in real time based on each child’s interim imaging and behavioral data, thereby closing the loop between measurement and treatment.

By advancing research along these five vectors, rooted in rigorous, pediatric-tailored methodology and clear translational pathways, we can move beyond descriptive neuroimaging toward interventions that dynamically adapt to each child’s developmental trajectory and clinical needs. Finally, by embracing technological advancements and fostering interdisciplinary collaboration, future research can refine our understanding of pediatric mood disorders and develop more effective, individualized treatment approaches.

## Figures and Tables

**Table 1:** Connections between brain functions and emotional/behavioral disorders.

Brain Area	Main Functions	Relevance in Disorders/Emotions
Prefrontal Cortex (PFC)	Emotion regulation, impulse control, long-term planning, strategic decision-making, stress management.	Controls the emotional responses generated by the limbic system; dysfunctions are associated with ADHD and alterations in bipolar disorder, where insufficient control can lead to exaggerated emotional responses.
Dorsolateral PFC (DLPFC)	Cognitive control, working memory, planning, and integration of complex emotional information.	Dysfunctions are linked to attention and self-regulation issues, often observed in ADHD and certain aspects of bipolar disorder.
Ventromedial PFC (VMPFC)	Emotion processing; decision-making based on values and rewards; integration with the amygdala, hippocampus, and nucleus accumbens.	Reduced control in this area can contribute to inappropriate or excessive emotional responses, affecting emotion regulation in bipolar disorder.
Orbitofrontal Cortex (OFC)	Integration and manipulation of sensory information, mediation of empathetic and socially appropriate responses, connectivity with the insula and ACC.	Dysfunctions can lead to compulsive behaviors and loss of emotional control, contributing to difficulties in modulating emotional responses.
Anterior Cingulate Cortex (ACC)	Mediation between emotions and cognitive processes, error monitoring, conflict resolution, management of physical and social pain.	Reduced ACC activity is associated with depression, difficulties in pain regulation, and problems in integrating emotions and executive functions, as observed in schizophrenia and bipolar disorder.
Insula	Integration of sensory, emotional, and cognitive signals; monitoring of bodily states; perception of pain; empathy; regulation of internal states.	Alterations in the insula are linked to dysfunctions in emotional perception, pain management, and certain compulsive behaviors and addictions; in bipolar disorder, its activity often correlates with symptom severity.
Limbic System	Processing of emotions, formation and retrieval of emotional memories, coordination of “fight or flight” responses.	An overactive amygdala can lead to exaggerated emotional responses; reductions in hippocampal volume and connectivity alterations may impair the contextualization of emotional experiences, as observed in bipolar disorder.
Cerebellum	Motor coordination, emotion regulation, identification and expression of emotions, support for empathy.	Lesions or anomalies in cerebellar–limbic connections can result in deficits in executive and emotional functions (e.g., cerebellar cognitive affective syndrome—CCAS) and have been associated with maladaptive behaviors and social difficulties in bipolar disorder.

**Table 2:** Brain imaging techniques.

Neuroimaging Technique	Description	Application
sMRI (Structural Magnetic Resonance)	Provides detailed images of the brain’s structure and morphology using strong magnetic fields and radiofrequency waves	Used to assess the anatomical structure of the brain, identifying abnormalities like tumors, strokes, and atrophy. It is also useful in monitoring neurodegenerative diseases and psychiatric disorders
fMRI (Functional Magnetic Resonance)	Measures brain activity by detecting changes in blood oxygenation levels (BOLD signals)	Used to map functional brain areas responsible for tasks like language, emotional regulation, and movement. It also assesses changes in brain function in psychiatric disorders like depression and anxiety
PET (Positron Emission Tomography)	Measures brain metabolic activity using radioactive tracers, highlighting areas of higher or lower activity	Used to study brain metabolism, showing activity in different brain regions. It is also used to assess neurotransmitter systems and understand neurochemical imbalances, and to evaluate neurodegenerative conditions like Alzheimer’s disease
DTI (Diffusion Tensor Imaging)	An MRI technique that allows the quantitative assessment of neuronal architecture by measuring the diffusion of water molecules along white matter pathways	Used to evaluate the integrity of white matter pathways. It is helpful in diagnosing and monitoring brain diseases like multiple sclerosis and strokes.

## Data Availability

No new data was created for this study.

## Abbreviations

The following abbreviations are used in this manuscript:

AI	Artificial Intelligence
WHO	World Health Organization
MRI	magnetic resonance
sMRI	structural magnetic resonance imaging
fMRI	functional magnetic resonance imaging
PET	positron emission tomography
DTI	diffusion tensor imaging
PFC	prefrontal cortex
DLPFC	dorsolateral prefrontal cortex
VMPFC	ventromedial prefrontal cortex
OFC	orbitofrontal cortex
ACC	anterior cingulate cortex
ADHD	attention deficit hyperactivity disorder
CCAS	cerebellar cognitive affective syndrome
BDNF	brain-derived neurotrophic factor
BOLD	blood-oxygen-level-dependent
CBT	cognitive behavioral therapy
RF-CBT	rumination-focused cognitive behavioral therapy
MDD	major depressive disorder
CBT-I	CBT for insomnia
CRS	circadian rhythm support
MBCT-C	mindfulness-based cognitive therapy for children
BMRM	body–mind relaxation meditation
MBI	mindfulness-based intervention
MRS	magnetic resonance spectroscopy
mPFC	medial prefrontal cortex
rt-fMRI-NF	real-time functional magnetic resonance imaging neurofeedback
PTSD	post-traumatic stress disorder
BPD	borderline personality disorder
PBD	pediatric bipolar disorder
BD	bipolar disorder
FA	fractional anisotropy
rTMS	repetitive transcranial magnetic stimulation
